# Uncommon Trimethoxylated Flavonol Obtained from* Rubus rosaefolius* Leaves and Its Antiproliferative Activity

**DOI:** 10.1155/2015/341216

**Published:** 2015-12-14

**Authors:** Marcel Petreanu, Emili Kamila Ferreira, Ana Paula M. Sagaz, Débora B. Vendramini-Costa, Ana Lúcia T. G. Ruiz, João Ernesto De Carvalho, Adriana Campos, Valdir Cechinel Filho, Franco Delle Monache, Rivaldo Niero

**Affiliations:** ^1^Programa de Pós-Graduação em Ciências Farmacêuticas, Núcleo de Investigações Químico-Farmacêuticas (NIQFAR), Universidade do Vale do Itajaí (UNIVALI), 88302-901 Itajaí, SC, Brazil; ^2^Centro Pluridisciplinar de Pesquisas Químicas, Biológicas e Agrícolas (CPQBA), Universidade Estadual de Campinas (UNICAMP), 13083-970 Campinas, SP, Brazil; ^3^Faculdade de Ciências Farmacêuticas, Universidade Estadual de Campinas (UNICAMP), 13083-859 Campinas, SP, Brazil

## Abstract

This study shows the evaluation the antiproliferative effect of the extract, fractions, and uncommon compounds isolated from* R. rosaefolius* leaves. The compounds were identified by conventional spectroscopic methods such as NMR-H^1^ and C^13^ and identified as 5,7-dihydroxy-6,8,4′-trimethoxyflavonol (**1**), 5-hydroxy-3,6,7,8,4′-pentamethoxyflavone (**2**), and tormentic acid (**3**). Both hexane and dichloromethane fractions showed selectivity for multidrug-resistant ovary cancer cell line (NCI-ADR/RES) with total growth inhibition values of 11.1 and 12.6 *μ*g/ml, respectively. Compound** 1** also showed selective activity against the same cell line (18.8 *μ*g/ml); however, it was especially effective against glioma cells (2.8 *μ*g/ml), suggesting that this compound may be involved with the* in vitro* antiproliferative action.

## 1. Introduction

Many are the advances in drug discovery for cancer prevention and treatment, but still there is a need for new therapies, as the statistics for cancer incidence and death by cancer worldwide are noteworthy [1]. Nature is an important source of new molecules with biological activities; around 75% of the anticancer agents are derived from or inspired by natural products [2].* Rubus* is the largest genus in the Rosaceae family, which is used especially as ornamental plants and for food, as it has tasty fruits. In the folk medicine, some species are used to treat different diseases, particularly diabetes [3, 4]. Phytochemical and pharmacological studies have shown that some species exert gastroprotective, antimicrobial, and cytotoxic effects, without genotoxic effects [5–11].* Rubus rosaefolius* is popularly known as “red berry,” and its fruits are consumed as food. Previous phytochemical investigations revealed the presence of sterols and terpenoids, which exhibited important antibacterial and antinociceptive activities [12, 13]. In this study, we have isolated and structurally characterized an uncommon methoxylated flavonol from the methanolic extract of* Rubus rosaefolius* leaves. Flavonols are an important class of compounds with many biological activities, including anticancer [14]. It is postulated that presence of 3-OH is essential for inhibition of topoisomerase in several cancer cell lines and substantially influences several mechanisms of antioxidant activity [14]. Also, methylation of some hydroxyl groups enhances the cytotoxic activity of some flavonols [14–16]. Bearing in mind the potential of methoxylated flavonols as anticancer agents and the need of new antiproliferative therapies, we evaluated the antiproliferative effect of the crude extract, fractions, and the uncommon methoxylated flavonol (**1**) in eight human cancer cell lines.

## 2. Results and Discussion

**Figure 1:**
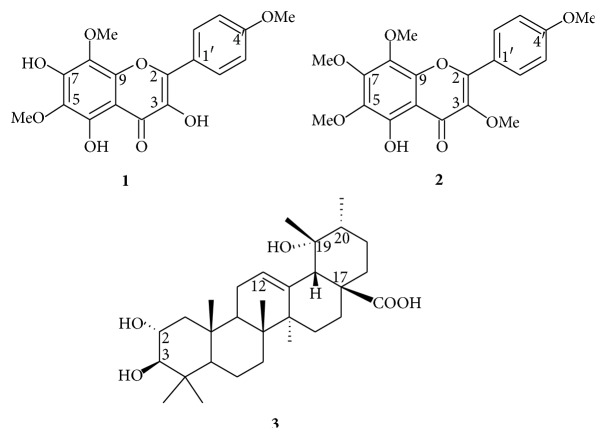
Molecular structure of compounds (**1**–**3**) obtained from* Rubus rosaefolius* leaves.

**Figure 2:**
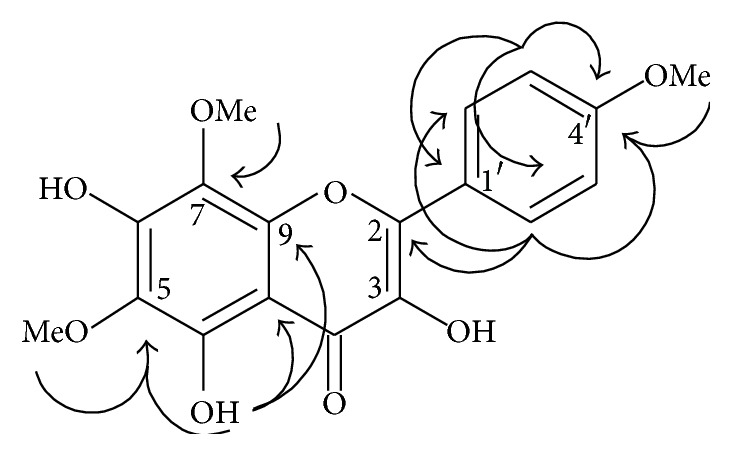
Molecular structure and significant long-range correlations observed in ^1^H-^13^C HMBC for compound** 1** obtained from* Rubus rosaefolius* leaves.

Dichloromethane (DCM) and hexane (HE) fractions of* R. rosaefolius* leaves were subjected to a series of silica gel chromatography columns to obtain three compounds, a uncommon methoxylated flavonol (**1**) along with the known compounds 5-hydroxy-3,6,7,8,4′-pentamethoxyflavone (**2**) and tormentic acid (**3**) (Figure 1). All compounds were identified by 1D and 2D RMN analysis and comparison of physical and spectroscopic data with those of literature [17, 18]. Compound** 1** was obtained as a yellow crystal and showed a [M]^+^ peak at* m/z* 360.3 in the electron impact ionization mass spectroscopy (EIMS), corresponding to a molecular formula C_18_H_16_O_8_. The IR spectrum showed the presence of hydroxyl groups (3354 cm^−1^), a conjugated carbonyl group (1624 cm^−1^), and aromatic rings (1500–1600 cm^−1^). The ^1^H NMR spectrum (Table 1) revealed three downfield singlets at *δ*H 12.26, 10.38, and 9.56, all disappearing by addition of D_2_O, suggesting the presence of three phenolic hydroxyls one of which chelated. Also displayed two* ortho* coupled doublets (2H each) at *δ*H 8.24 and *δ*H 7.17 suggesting * p*-substituted aromatic rings, and three methoxyl groups at *δ*H 3.79 (3H), 3.80 (3H), and 3.85 (3H). The ^13^C NMR data and HMBC correlations in DMSO-*d6* are reported in Table 1 and indicated that the protons at 8.24 (H-2′,6′) correlated with the carbons at *δ* 129.2 (C-2′,6′), 146.3 (C-2), and 160.5 (C-4′). Similarly, protons at 7.17 (d, *J* = 8.7 Hz, H-3′5′) correlated with carbons at *δ* 114.2 (C-3′5′), 123.4 (C-1′), and 160.5 (C-4′), confirming a* p*-substituted B ring. Methoxylic protons observed at *δ* 3.85, 3.80, and 3.79 showed correlations with aromatic carbons at *δ* 127.7 (C-8), 131.0 (C-6), and 160.5 (C-4′), respectively, demonstrating that the methoxylic groups were located at C-6 and C-8 of ring A and C-4′ of ring B (Figure 2). All the data and comparison with those reported previously and combined with other partial structures, enabled this compound to be identified as 5,7-dihydroxy-6,8,4′-trimethoxyflavonol (**1**). At the best of our knowledge, compound** 1** was obtained by synthesis [19] and isolated only once as natural product [20].

Initially, we evaluated the antiproliferative activity of the crude extract against four cell lines such as U251, MCF7, NCI-H460, and 786-0. The concentrations which inhibited 50% cell growth were 28.9, 29.6, 36.1, and 225.2 *μ*g/mL, respectively. Considering these results, fractions and compound** 1** were tested for their antiproliferative activity against eight human cancer cell lines and a nontumor cell line in four concentrations (0.25, 2.5, 25, and 250 *μ*g/mL), using doxorubicin as positive control. Results are summarized in Table 2 and were expressed as the concentration required for completely inhibiting the cancer cell line growth after 48 hours of exposure to the samples (total growth inhibition, TGI).

As shown in Table 2, DCM and HE fractions demonstrated antiproliferative activity for most of the human cancer cell lines evaluated. DCM fraction was more potent than HE fraction, showing selectivity for breast (MCF-7), multidrug-resistant ovary carcinoma (NCI-ADR/RES), kidney (786-0), and colon (HT-29), with TGI (total grown inhibition) values of 17.8, 11.1, 19.3, and 24.4 *μ*g/mL, respectively. On the other hand, ethyl acetate fraction (EA) did not demonstrate significant activity in any of the concentrations tested. Regarding the anticancer activity of hexane fraction, it can be partially attributed to compound** 3**, which is reported to have a potent free radical scavenging ability and considerable antiproliferative activity against MCF-7 and HepG2 cell lines [21]. Considering that compounds** 1** and** 2** were isolated from DCM fraction and that there are already reports of the anticancer activity of tormentic acid and its derivatives [22, 23], only compound** 1** was evaluated against the same cell lines. As can be observed, compound** 1** demonstrated* in vitro* antiproliferative effects for glioma (U251), multidrug-resistant ovary carcinoma (NCI-ADR/RES), kidney (786-0), lung (NCI-H460), ovary (OVCAR-3), and leukemia cells (K-562), with TGI values of 2.8, 18.8, 15.8, 14.1, 14.5, and 17.5 *μ*g/mL, respectively (Table 2). Despite being less potent than DCM fraction for breast cancer cell line (MCF-7), the isolated compound exhibited better* in vitro* antiproliferative activity, being especially potent for glioma, with a TGI value close to that of the positive control, doxorubicin. Glioblastoma multiform (GBM) is the most common primary tumor of the central nervous system (CNS), and its occurrence in older age groups has increased in recent decades, with treatment or cure presenting a challenge to the scientific community [24].

Although extract and fractions antiproliferative effect may be attributed to the presence of several compounds, these results suggest that this uncommon methoxylated flavonol may be more closely involved with the* in vitro* anticancer activity. Although some studies have demonstrated the anticancer activity of* Rubus* genus, no reports have been found for* R. rosaefolius*, this study being the first one to describe the antiproliferative activity of this specie.

## 3. Material and Methods

### 3.1. General

IR spectra were recorded on a BOMEM-100 with Fourier-Transform Infrared (FT-IR, BOMEM, St. Jean Baptiste, QB, Canada) spectrometer using KBr. EIMS were recorded on Shimadzu Gas Chromatograph (QP-2010S series, Kyoto, Japan) coupled with a mass spectrometric detector equipped with a NIST08 software database. 1D and 2D NMR spectra were recorded on a Bruker AV-300 (Bruker, Karlsruhe, Germany) in DMSO-*d6*. Chemical shifts (*δ*) are expressed in ppm, and coupling constants are given in Hz.

### 3.2. Chromatographic Conditions

Recoated aluminum plates silica gel 60F_254_ (E. Merck, Darmstadt, Germany) for TLC were used and visualized under UV at 254 and 366 nm and by spraying with anisaldehyde sulfuric and FeCl_3_ reagents. Column silica gel 70–230 mesh and flash silica gel 230–400 mesh (E. Merck, Darmstadt, Germany) were used for the column chromatography. Melting points were determined on a Micro-Química APF-300 apparatus (Micro-Química, Florianópolis, SC, Brazil) and are uncorrected. The purity of the isolated compound was examined by thin layer chromatography (TLC) using Merck silica gel precoated aluminum plates (thickness = 200 *μ*m) and several solvent systems of different polarities. All the reagents and solvents used were of analytical grade and were purchased from Sigma-Aldrich Co. (St. Louis, MO, USA) and Merck KGaA (Darmstadt, Germany).

### 3.3. *In Vitro* Anticancer Activity Assay

Human tumor cell lines, U251 (glioma), MCF-7 (breast), NCI-ADR/RES (multidrug-resistant ovary), 786-0 (kidney), NCI-H460 (lung, non-small cells), OVCAR-3 (ovary), HT-29 (colon), and K-562 (leukemia) were kindly provided by the United States National Cancer Institute (NCI). Nontumor cell line HaCat (human keratinocytes) was donated by Professor Dr. Ricardo Della Coletta, FOP/UNICAMP. Stock cultures were grown in medium containing 5 mL of RPMI 1640 (GIBCO BRL) supplemented with 5% fetal bovine serum (GIBCO BRL). Penicillin (100 U/mL) and streptomycin (100 *μ*g/mL) were added to experimental cultures. Cells in 96-well plates (100 *μ*L cells well-1) were exposed to sample concentrations in DMSO/RPMI (0.25, 2.5, 25, and 250 *μ*g mL^−1^) at 37°C, 5% of CO_2_ in air for 48 h. Final DMSO concentration did not affect cell viability. Doxorubicin hydrochloride (0.1 mg/mg; Europharma) was adopted as a positive control (0.025, 0.25, 2.5, and 25 *μ*g/mL). Afterwards, cells were fixed with 50% trichloroacetic acid and cell proliferation was determined by spectrophotometric quantification (540 nm) of cellular protein content using sulforhodamine B assay [25]. In this assay, the cell population density is measured at time zero (when samples are added) thus allowing the calculation of the concentrations that promote total growth inhibition (TGI). These concentrations are calculated from T = T0, in which the amount of protein at the end of sample incubation (T) is equal to the amount at the beginning (T0) [26]. Using the concentration-response curve for each cell line, TGI (total growth inhibition) was determined through nonlinear regression analysis using software ORIGIN 8.0 (OriginLab Corporation) [26]. Experiment was conducted in triplicate.

### 3.4. Plant Material

The* R. rosaefolius* plant material was collected in the town of Itajaí, SC, Brazil (26° 54′ 28′′ S; 48° 39′ 43′′ W) in March 2013 and identified by Dr. Ademir Reis (Botanical Department of the Federal University of Santa Catarina). A voucher specimen was deposited at the Barbosa Rodrigues Herbarium (Itajaí-SC) under number V.C. Filho 035.

### 3.5. Extraction and Isolation

Air-dried powdered leaves (580 g) of* R. rosaefolius* were exhaustively extracted with methanol (MeOH) at room temperature for seven days. The macerates were filtered and concentrated under reduced pressure in a rotatory evaporator, yielding 174.0 g of crude methanol extract. The extract was suspended in MeOH-H_2_O (90 : 10) mixture and subjected to liquid-liquid partition using solvents of increasing polarity such as HE, DCM, and EA. Part of the DCM fraction (4.5 g) was subjected to column chromatography over silica gel and eluted with DCM-MeOH (100 : 0-0 : 100) in increasing order of polarity, yielding 164 fractions which were combined based on TLC profiles. Fraction 31–41 (1.0 g) was rechromatographed using the same solvent system, yielding new 99 subfractions, which were pooled according to their similar chromatographic profiles. Subfractions 14–28 were then eluted with a mixture of Hexane : Acetone (75 : 25), yielding 35 subfractions in which subfractions 10–30 presented as a yellow crystal (35 mg) and were identified as 5,7-dihydroxy-6,8,4′-trimethoxyflavonol (**1**) by NMR, DEPT, HMBC, IR data in comparison with those reported previously [19, 27]. Also in relation to DCM fraction isolation, fractions 100–123 (985 mg) from first column were combined and rechromatographed using DCM-MeOH (100 : 0-0 : 100) as solvent system, yielding 125 subfractions, which were pooled according to their similar chromatographic profiles. Subfractions 45–47 presented as a pure colorless crystal (18 mg) and were identified as tormentic acid (**3**) based on IR, Mass, and NMR data in comparison with those reported previously [18]. Similarly, part of the HE fraction (9.04 g) was subjected to column chromatography over silica gel and eluted with DCM-MeOH (100 : 0-0 : 100) in increasing order of polarity to afford 123 fractions, which were combined based on their TLC profiles. Fraction 49–68 (2.12 g) was rechromatographed using the same solvent system, yielding new 105 subfractions. Of these, fraction 69–71 (14.0 mg) was rechromatographed using the same solvent system, yielding new 20 subfractions. Subfractions 1–6 presented as a yellow crystal (4.7 mg) and were identified as 5-hydroxy-3,6,7,8,4′-pentamethoxyflavone (**2**) based on IR, Mass, and NMR data in comparison with those reported previously [17].

#### 3.5.1. 5,7-Dihydroxy-6,8,4′-trimethoxyflavonol (**1**)

Yellowish solid; mp 295–297°C; IR (KBr) *λ*_max_ 3354, 2970, 1664, 1629, 1220 cm^−1^; ^1^H NMR (300 MHz, DMSO-*d6*) *δ* 12.26 (1H,* s*, C-7-OH); 10.38 (1H,* s*, C-5-OH); 9.56 (1H,* s*, C-3-OH); 8.24 (2H,* d*, *J* = 9 Hz, H-2′6′); 7.17 (2H,* d*, *J* = 9 Hz, H-3′5′); 3.85 (3H,* s,* C-8-OMe); 3.80 (3H,* s*, C-4′-OMe); 3.79 (3H,* s*, C-6-OMe); ^13^C NMR (75 MHz, DMSO-*d6*) *δ* 176.4 (C=O, C-4), 160.5 (C-4′), 150.6 (C-7), 147.6 (C-5), 146.3 (C-2), 144.5 (C-9), 135.8 (C-3), 131.0 (C-6), 129.2 (C-2′6′), 127.7 (C-8), 123.4 (C-1′), 114.2 (C-3′5′), 102.4 (C-10), 61.1 (C-8-OMe), 60.2 (C-6-OMe), 55.4 (C-4′-OMe); EIMS* m/z* 360.3 [M]^+^ (calcd for C_18_H_16_O_8_: 360.3148).

#### 3.5.2. 5-Hydroxy-3,6,7,8,4′-pentamethoxyflavone (**2**)

Yellowish solid; mp 119–123°C; IR (KBr) *λ*_max_ 3241, 2985, 1752, 1546, 1239 cm^−1^; ^1^H NMR (300 MHz, CDCl_3_) *δ* 8.17 (2H,* d*, *J* = 9 Hz, H-2′6′); 7.06 (2H,* d*, *J* = 9 Hz, H-3′5′); 4.10 (3H,* s*, C-7-OMe); 3.95 (6H,* s*, C-6-OMe, C-8-OMe) 3.90 (3H,* s*, C-4′-OMe); 3.87 (3H,* s,* C-3-OMe); ^13^C NMR (75 MHz, CDCl_3_) *δ* 179.3 (C=O, C-4), 161.9 (C-4′), 156.1 (C-2), 152.9 (C-7), 149.2 (C-5), 144.9 (C-9), 138.6 (C-3), 136.1 (C-6), 132.4 (C-8), 130.3 (C-2′6′), 122.8 (C-1′), 114.3 (C-3′5′), 107.5 (C-10), 62.1 (C-8-OMe), 61.7 (C-7-OMe), 61.2 (C-6-OMe), 60.1 (C-3-OMe), 55.4 (C-4′-OMe); EIMS* m/z* 388.1 [M]^+^ (calcd for C_20_H_20_O_8_: 360.3680).

#### 3.5.3. Tormentic Acid (**3**)

White powder; mp 269–275°C; IR (KBr) *λ*_max_ 3450, 2900, 1688, 1500, 1100 cm^−1^; ^1^H NMR (300 MHz, CD_3_OD): *δ* 5.29 (1H,* s*, H-12), 4.62 (1H,* br s*, 19-OH), 3.66 (1H,* dd*, *J* = 11.1, 4.5 Hz, H-2), 2.93 (1H,* d*, *J* = 9.3 Hz, H-3), 2.50 (1H,* s*, H-18), 1.35 (3H,* s*, H-27), 1.19 (3H,* s*, H-29), 1.02 (3H,* s*, H-23), 1.00 (3H,* d*, *J* = 6.4 Hz, H-30), 0.94 (3H,* s*, H-26), 0.81 (3H,* s*, H-24), 0.79 (3H,* s*, H-25); ^13^C NMR (75 MHz, CD_3_OD) 182.4 (C28), 140.2 (C-13), 129.4 (C-12), 84.7 (C-3), 73.7 (C-19), 69.7 (C-2), 56.8 (C-5), 55.2 (C-18), 48.6 (C-9), 48.3 (C-1), 43.2 (C-20), 42.7 (C-14), 41.2 (C-8), 40.7 (C-4), 39.4 (C-22), 39.2 (C-10), 34.2 (C-7), 29.7 (C-15), 29.4 (C-23), 27.4 (C-21), 27.2 (C-30), 26.7 (C-16), 25.0 (C-27), 24.9 (C-11), 19.8 (C-6), 17.6 (C-24), 17.6 (C-26), 17.2 (C-25), 16.8 (C-29); EIMS* m/z*: 488.2 [M]^+^ (calcd for C_30_H_48_O_5_: 488.6991).

## 4. Conclusions

In summary, our results showed the presence of a uncommon methoxylated flavonol identified as 5,7-dihydroxy-6,8,4′-trimethoxyflavonol along with two known compounds, 5-hydroxy-3,6,7,8,4′-pentamethoxyflavone and tormentic acid. They also showed that the methanolic extract, hexane, and dichloromethane fractions presented an interesting* in vitro* antiproliferative profile. Trimethoxylated flavonol isolated from* Rubus rosaefolius* leaves also showed promising antiproliferative profile, especially against glioma cell line, making this compound of great interest in the search for new chemotherapeutic agent.

## Figures and Tables

**Table 1:** ^1^H and ^13^C NMR data of compound  **1** in DMSO-*d6*.

Position	^1^H NMR	^13^C NMR	HMBC
2		146.3	
3		135.8	
4		176.4	
5		147.3	
6		131.0	
7		150.6	
8		127.7	
9		144.5	
10		102.4	
1′		123.4	
2′, 6′	8.24 d (9.0)	129.2	C-2, C-2′6′, C-4′
3′, 5′	7.17 d (9.0)	114.2	C-1′, C-3′5′, C4′
4′		160.5	
OMe-4′	3.80 s	55.4	C-4′
OMe-6	3.79 s	60.2	C-6
OMe-8	3.85 s	61.1	C-8
OH-3	9.56 s		
OH-5	10.38 s		
OH-7	12.26 s		C-6, C-9, C-10

*δ* in PPM; *J* in Hz; ^1^H NMR at 300 MHz and ^13^C NMR at 75 MHz.

**Table 2:** Antiproliferative activity of doxorubicin, hexane (HE), dichloromethane (DCM), and ethyl acetate (EA) fractions and compound **1** against human cancer cell lines^a^.

Total growth inhibition (TGI) (*µ*g/mL)^b^					
Cell lines	Doxorubicin	HE	DCM	EA	Compound **1**
Glioma (U251)	4.1	35.4	51.1	>250	2.8
Breast (MCF-7)	0.62	45.2	17.8	>250	55.8
Multidrug-resistant ovary carcinoma (NCI-ADR/RES)	9.1	12.6	11.1	>250	18.8
Kidney (786-0)	0.46	30.7	19.3	>250	15.8
Lung, non-small cells (NCI-H460)	4.3	56.3	135.0	>250	14.1
Ovary (OVCAR-3)	2.4	46.1	89.7	>250	14.5
Colon (HT-29)	1.3	28.2	24.4	>250	>250
Leukemia (K-562)	>25	>250	250.0	>250	17.5
Nontumoral keratinocyte (HaCat)	0.79	10.4	25.8	>250	21.4

^a^Assessed by the sulforhodamine B (SRB) assay. ^b^TGI values represent the concentration necessary (*µ*g/mL) for total inhibition of cancer cell proliferation. Values were determined through nonlinear regression analysis using the ORIGIN 8.0 (OriginLab Corporation). Dose range tested: 0.25 to 250 *µ*g/mL. Experiment was conducted in triplicate.
